# Adults With Intellectual Disabilities and Incontinence: Assessment and Toileting Issues

**DOI:** 10.1111/jir.13202

**Published:** 2024-11-24

**Authors:** Janet Finlayson, Dawn A. Skelton, Paul Ord, Fiona Roche, Audrey Marshall, John Butcher, Nick Gore

**Affiliations:** ^1^ School of Health and Life Sciences, Research Centre for Health (ReaCH) Glasgow Caledonian University Glasgow UK; ^2^ NHS Greater Glasgow and Clyde Glasgow UK; ^3^ Tizard Centre University of Kent in Canterbury Canterbury UK

**Keywords:** assessment, bowel incontinence, faecal incontinence, intellectual disability, toileting issues, urinary incontinence, urinary tract infections

## Abstract

**Background:**

Urinary and bowel incontinence are more common in adults with intellectual disability (ID), compared to the general population. Little is known about their incontinence experiences and toileting issues. The aim was to learn about their experiences and toileting issues.

**Method:**

Incontinence and toileting issues assessment was conducted with a community‐based sample of 22 adults with ID and urinary incontinence, with or without bowel incontinence. Assessment included the *IPSS*, *ICIQ‐UI*, and *POTI* checklists; bladder scans; and urine sample screening for presence of a urinary tract infection.

**Results:**

The majority (19 adults, 86%) developed urinary incontinence during adulthood. Seven adults (32%) also experienced bowel incontinence, and constipation was the most commonly reported health condition (13 adults, 59%), other than urinary incontinence. Fifty per cent (11 adults) had been treated for a urinary tract infection within the previous 12 months.

**Conclusion:**

There is an urgent need to develop accessible and reliable incontinence assessment materials with and for adults with ID and their supporters. These assessments should pay close attention to health conditions that can cause incontinence in this group and factors associated with incontinence which are more commonly experienced by adults with ID. These factors are potentially modifiable.

## 1 Introduction

Urinary incontinence (UI; involuntary loss of urine) is a common problem, which affects between 2% and 36% of the general population (Milsom et al. 2014). Variation in prevalence rates is dependent upon two main risk factors, being female (e.g., due to child‐bearing) and advancing age (e.g., due to functional decline) (Milsom and Gyhagen 2019; Milsom et al. 2014). Other risk factors include obesity, urinary tract infection (UTI), certain medications (e.g., anticholinergics, antihistamines and antipsychotics) (Dobrek 2023), constipation and fluid intake (Bardsley 2016). The different types of UI are stress, urge, mixed, overflow, nocturnal, reflex and functional incontinence. These types of UI have differing causes and symptoms (Bardsley 2016).

Approximately 1 in every 12 adults (8%) experience bowel incontinence (BI; involuntary loss of faeces) (Mack et al. 2023). Risk factors for BI also include being female and advancing age, as well as conditions such as irritable bowel syndrome (IBS) (Mack et al. 2023).

UI and BI are more common amongst adults with intellectual disabilities (IDs). Previous research has demonstrated that between 26% and 52% of adults with ID have UI (due to variation in, e.g., sampling methods), with higher prevalence rates closely associated with lower adaptive functioning (Van Timmeren et al 2016; De Waal et al 2009). A population‐based study of 511 adults with ID (aged 18 years and over) found that 166 adults (32%) had UI and 118 adults (23%) had BI (Finlayson et al. 2010). UI has also been shown to be a risk factor for falls (Finlayson et al. 2010), mental ill health, and behaviours, which others may view as challenging in adults with ID (Cooper et al. 2007). A population‐based study of older adults with ID (aged 40 years and over) also reported a UI prevalence of 28%, which was three times higher than the 9% prevalence in a comparable general population sample (Keenan et al. 2018; McCarron, Swinburne, and Burke 2011).

Despite incontinence, which impacts on health and wellbeing, being a common amongst adults with ID, there is a paucity of evidence on the characteristics or types of incontinence they experience and their toileting issues. One previous study conducted portable ultrasound bladder scans with 346 adults with ID and found that 30 (9%) were experiencing postvoid urine retention (PVUR) (De Waal et al 2009). In terms of toileting issues, studies conducted by Matson, Horovitz, and Sipes (2011a) and Matson et al. (2011b) in the United States resulted in the development of a 30‐item *Profile of Toileting Issues (POTI) Checklist* adapted for use with people with ID, but use of the POTI checklist more widely with adults with ID has not been reported in the literature.

The aim of this research was to conduct incontinence and toileting issues assessments with a community‐based sample of adults with ID with UI, who may or may not also have BI, to assess the types and characteristics of incontinence, and toileting issues, they are experiencing.

## 2 Method

### 2.1 Participants and Process

Adults with ID and incontinence were recruited via project information sheets, provided by their community ID health care professional working in teams across National Health Service (NHS) Greater Glasgow & Clyde and NHS Lanarkshire. This purposive sample was recruited as part of a wider study, to develop personalised toileting plans with adults with ID (Finlayson, Gore, and Skelton 2024).

Community ID health care professionals were asked to distribute the project information to any of their clients with ID on their caseloads who met the following inclusion criteria: Person has UI (with or without BI), and person is able to sit on a toilet for up to 3 min, with or without support. The latter was necessary for the intervention stage of this project.

Adults with ID and incontinence who chose to take part, with support from their supporter (relative or support worker), were then visited at home by a researcher, to complete the consent process and conduct a research interview. Toileting assessment was completed with 22 adults with ID with incontinence. Their characteristics and health status are described in Table 1.

**TABLE 1:** : Participants' characteristics and health status.

Characteristic	*N* = 22 (100%)
Age	Mean 43 years Median 38 years Range 18–71 years Standard deviation (SD) 16.9 years
Sex	Male 9 (41%) Female 13 (59%)
Ethnicity	Caucasian 22 (100%)
Accommodation type	Lives alone, independently 2 (9%) Lives with family 12 (54%) Supported living—individual tenancy 3 (14%) Supported living—group tenancy 5 (23%)
Cause of ID	Down syndrome 2 (9%) Birth injury 3 (14%) Unknown 17 (77%)
Level of ID	Mild 2 (9%) Moderate 10 (46%) Severe 8 (36%) Profound 2 (9%)
Are you autistic?	Yes 8 (36%) No 14 (64%)
Mobility level	Walks independently 12 (54%) Walking stick or frame 5 (23%) Wheelchair user (indoors only) 3 (14%) Wheelchair user (indoors and outdoors) 2 (9%)
Do you have a visual impairment?	Yes 6 (27%) No 15 (69%) Don't know 1 (4%)
Do you have a hearing impairment?	Yes 3 (14%) No 18 (82%) Don't know 1 (4%)
Number of diagnosed health conditions	Mean 5 Median 4 Range 1–10 SD 1.8
Known health conditions	Urinary incontinence 22 (100%) Constipation 13 (59%) Bowel incontinence 7 (32%) Anxiety 5 (23%) Epilepsy 4 (23%) Cerebral palsy 4 (23%) Diabetes type 2 4 (18%) Depression 3 (14%) Asthma 3 (14%) Hypertension 2 (9%) History of colon cancer 1 (4%) History of breast cancer 1 (4%) Long QT syndrome 1 (4%) Bicuspid aortic valve 1 (4%) Schizophrenia 1 (4%) Obsessive compulsive disorder 1 (4%) Basal ganglia calcification 1 (4%) Cauda equina syndrome 1 (4%) Parkinson's disease 1 (4%) Hyperparathyroidism 1 (4%) Hypothyroidism 1 (4%) Hidradenitis suppurative 1 (4%) Psoriasis 1 (4%) Arthritis 1 (4%) Dysphasia 1 (4%) Irritable bowel syndrome 1 (4%) Oesophagitis reflux 1 (4%) Cystic kidney disease 1 (4) Granulomatosis 1 (4%) Right hemiplegia 1 (4%) Cerebellar ataxia 1 (4%) Osteoporosis 1 (4%)
Number of prescribed drugs	Mean 6 drugs Median 6 drugs Range 0–13 drugs SD 3.7
Prescribed drugs with possible side effects	
Yes	12 (54%)
Chlorpromazine	1 (4%)
Risperidone	2 (9%)
Fluoxetine	4 (18%)
Tegretol	1 (4%)
Sertraline	5 (23%)
Chloraphenamine	1 (4%)

Five (23%) adults with ID were able to use a toilet on their own independently, 12 (54%) required support from another person to use a toilet and 5 (23%) required support from another person and the use of an aid (e.g., raised toilet seat) to use a toilet. When in public places, 15 (68%) needed to use an accessible toilet for people with disabilities, 2 (9%) needed to use a changing places facility (http://www.changing‐places.org) and 5 (23%) used a regular public toilet.

Sixteen (73%) adults with ID used pads for their incontinence, two of whom also used a bed mat for protection during the night.

### 2.2 Ethical Approval

Ethical approval was granted by the national Scotland A research ethics committee. Written consent was obtained from seven participants with ID and 15 nearest relatives on the participants' behalf. All participants were able to withdraw from the study at any time, if they chose to do so, and all were encouraged to participate with assistance from a supporter who knew them well, to ensure their rights, wishes, responses and willingness to continue or not was observed and respected at all times.

### 2.3 Project Materials

The accessible project information and consent forms, which included easy language, pictures and symbols, were developed by the research team and reviewed by two parents of people with ID. The home visit research interview schedule was developed by the research team, to collect personal and demographic information, as well as information about the person's incontinence and toileting issues. The schedule included both closed and open questions. A copy of all project materials is available from the authors.

The *International Prostate Symptom Score* (IPSS) (Barry et al. 1992) to score any urinary tract symptoms and the *International Consultation on Incontinence Questionnaire – Urinary Incontinence* (ICIQ‐UI) (Avery et al. 2004) to assess severity of any UI symptoms and their impact on quality of life were completed with each participant. Both tools are valid and reliable measures, which are used widely with men and women across clinical and research settings (Booth et al. 2018; Lim et al. 2017). The *Profile of Toileting Issues* (POTI) *Checklist* (Matson, Horovitz, and Sipes 2011a) was also completed with each participant.

Participants, with their supporters, were asked to complete daily fluid intake charts over the next 72 h. Each participant was also invited to provide a urine sample, to screen for a UTI, and bladder scans to screen for weak bladder, and PVUR.

Urine samples were collected in specimen containers, then returned to the university laboratory for immediate analysis, to screen for the presence of a UTI. Urine sample results were categorised according to current UK standards for investigating urine (Public Health England 2019). A portable *Caresono* bladder scanner was used to scan the person's bladder twice: prevoiding to measure bladder volume and postvoiding to check for PVUR. A healthy bladder volume for adults is between 300 and 400 mL (Lukacz et al. 2011). Bladder retention over 200 mL indicates inadequate emptying, over 300 mL is suggestive of PVUR and over 400 mL is considered PVUR (Ballstaedt, Leslie, and Woodbury 2024).

### 2.4 Analysis

Data were entered on to an IBM SPPSS version 28 dataset, which was used to generate frequency and descriptive statistics. No inferential statistical analysis was conducted due to the small sample size. All numbers were rounded to the nearest whole number so may not add up to 100% in all cases.

Qualitative data collected via open‐ended questions were entered on to the same dataset, as string variables, and subject to content analysis, to count and order the responses into categories.

## 3 Results

### 3.1 Urine Incontinence Assessment, UTIs, and Daily Fluid Intake

The participants' UI assessments are detailed in Table 2.

**TABLE 2:** Urinary incontinence assessment results

Assessment	*N* = 22
Number of years have experienced urinary incontinence	Mean 8 years Median 5 years Range 5 months to 34 years Standard deviation 9.27
Has your urinary incontinence changed over time?	Yes 17 (77%)
*Has become more frequent*	*7 (32%)*
*Physical support required to use toilet has increased*	*6 (27%)*
*Has developed anxiety around visiting a toilet*	*1 (4%)*
*Now uses pads during the night as well*	*1 (4%)*
*Less able to recognise and communicate toilet need*	*1 (4%)*
Reduced bladder sensation	1 (4%)
	No 5 (23%)
Do you experience a sensation or urge to use the toilet?	Yes 20 (92%) No 1 (4%) Don't know 1 (4%)
Do you experience any pain or discomfort passing urine?	Yes 8 (37%)
*Stinging sensation*	6 (27%)
*Stomach cramps*	3 (14%)
	No 14 (63%)
Daily fluid intake (based on 3‐day average)	Mean 1919 mL Median 1978 mL Range 770 mL to 3000 mL Standard deviation 663 mL
Most common type of drink over the 72‐h assessment period:	
Cordial/squash	8 (36%)
Tea	4 (18%)
Carbonated soft drink (e.g., cola)	3(14%)
Water	2 (9%)
Coffee	1 (4%)
Milk	1 (4%)
Fruit tea	1 (4%)
*Missing*	*2 (9%)*
Did you drink any alcohol over the 72‐h assessment period?	No 20 (91%) *Missing 2 (9%)*
**IPSS:** Over the past month, how often have you had a sensation of not emptying your bladder completely after you finish urinating?	Almost always 3 (14%) Less than half the time 1 (4%) Not at all 6 (27%) Don't know 12 (55%)
**IPSS:** Over the past month, how often have you had to urinate again less than 2 h after you finished urinating	Almost always 7 (32%) More than half the time 3 (14%) About half the time 3 (14%) Less than half the time 1 (4%) Not at all 1 (4%) Don't know 7 (32%)
**IPSS:** Over the past month, how often have you found you stopped and started again several times when you urinated?	Almost always 2 (9%) More than half the time 1 (4%) Less than half the time 2 (9%) Not at all 12 (55%) Don't know 5 (23%)
**IPSS:** Over the past month, how difficult have you found it to postpone (delay or hold) urination?	Almost always 8 (36%) More than half the time 4 (18%) About half the time 2 (9%) Less than half the time 1 (4%) Not at all 3 (14%) Don't know 4 (18%)
**IPSS:** Over the past month, how often do you have a weak urinary stream (flow)?	Almost always 2 (9%) Not at all 19 (87%) Don't know 1 (4%)
**IPSS:** Over the past month, how often have you had to push or strain to begin urination?	Almost always 2 (9%) Not at all 19 (87%) Don't know 1 (4%)
**IPSS:** Over the past month, how many times did you typically urine during the night (from when you went to bed until the time you got up in the morning)?	5 times or more 1 (4%) 3–4 times 7 (32%) 1–2 times 6 (27%) None 5 (23%) Don't know 3 (14%)
**ICIQ‐UI:** How often do you leak urine?	All the time 1 (4%) Several times a day 7 (32%) About once a day 5 (23%) Once a week or less 1 (4%) Never 3 (14%) Don't know 3 (14%)
**ICIQ‐UI:** How much urine do you usually leak (whether you wear protection or not)?	A large amount 1 (4%) A moderate amount 4 (18%) A small amount 7 (32%) None 3 (14%) Don't know 7 (32%)
**ICIQ‐UI:** How much does leaking urine interfere with your daily life (Likert scale between 0 [*not at all*] and 10 [*a great deal]*)	Mean 7 Median 7 Range 0–10 Standard deviation 2.9
**ICIQ‐UI:** Never leaks urine	Yes 3 (14%) No 19 (86%)
**ICIQ‐UI:** Leaks before you can get to the toilet	Yes 1 (4%w) No 21 (96%)
**ICIQ‐UI:** Leaks when you cough or sneeze	Yes 2 (9%) No 20 (91%)
**ICIQ‐UI:** Leaks when you are asleep	Yes 1 (4%) No 21 (96%)
**ICIQ‐UI:** Leaks when you are physically active/exercising	Yes 1 (4%) No 21 (96%)
**ICIQ‐UI:** Leaks all the time	Yes 4 (18%) No 18 (82%)

*Note:* Descriptions of responses elicited from addition open‐ended questions (e.g., ‘Please describe …’) provided in italics may not add up to the number of participants who answered yes or no.

Eleven adults (50%) had been diagnosed with a UTI within the previous 12 months, 7 (32%) more than once. Of 20 adults screened during this assessment (2 declined), 10 (50% of 20 adults) were also found to have a possible or probable UTI. This study's UTI screening procedure, results and subsequent follow‐up by the person's general practitioner as necessary have been published separately (Finlayson et al. 2025).

The mean daily fluid intake of 20 participants with ID (2 did not complete fluid charts) was 1919 mL (ml) (ranging from 770 to 3000 mL, standard deviation 663 mL). Seven adults with ID (32%) were drinking less than the recommended 1500–2000 mL on average per day, 3 (14%) were drinking within that range, 10 (45%) were drinking in excess of the recommended amount, and 2 (0%) did not provide fluid chart data. Caffeinated and alcoholic drinks were not widely consumed by this sample.

### 3.2 Bladder Scans

Only six (27%) of the adults with ID opted to complete a bladder scan. Of these six adults, the mean volume of their bladder prevoiding was 258 mL, and the mean volume of their bladder postvoiding was 42 mL. Of the 16 (73%) who declined a bladder scan, only 3 gave a reason why: 1 person already had a recent scan with a nurse (and was found to have PVUR); 1 person could not urinate on demand; and 1 person because there was no female supporter present to chaperone.

### 3.3 Bowel Assessment and Bowel Incontinence

Participants' bowel assessments are presented in Table 3. Seventeen (77%) adults with ID reported that they have a bowel movement every day, and only two adults did not know when they needed to use the toilet to pass a bowel movement (they did not experience a sensation or urge). Thirteen (59%) adults with ID were prescribed medication for constipation (Table 1).

**TABLE 3:** Bowel assessment.

Item discussed	*N* = 22
How often do you have a bowel movement?	Every day 17 (77%) 2–3 days per week 2 (9%) 4–6 days per week 2 (9%) Don't know 1 (5%)
Do you know when you need to pass a bowel movement? (Do you experience a sensation or urge?)	Yes 18 (82%) No 4 (18%)
Do you experience pain or discomfort around using the toilet to pass a bowel movement?	Yes 7 (32%) *Pain (4 people)* *Stinging sensation (2 people)* *Abdominal cramps (2 people)* No 13 (59%) Don't know 2 (9%)
Do you experience passing hard faeces?	Yes, at least weekly 9 (41%) Yes, at least monthly 6 (27%) No 7 (32%)
Do you experience faecal impaction? (Build‐up of hard faeces with possible overflow or liquid faeces)	Yes 2 (9%) No 16 (73%) Don't know 4 (18%)
Do you experience faecal leakage? (Involuntary loss of faeces on physical exertion, sneezing, and coughing)	Yes, at least daily 2 (9%) Yes, at least weekly 1 (5%) Yes, at least monthly 1 (5%) Yes, less than monthly 2 (9%) No 16 (73%)
Do you experience frequent and urgent defecation? (Three or more times during the day with severe urgency and usually loose faeces)	Yes, at least daily 2 (9%) Yes, at least weekly 2 (9%) Yes, less than monthly 1 (5%) No 17 (77%)

Seven (32%) of the adults with ID also have bowel incontinence. Their BI is described in table 4.

**TABLE 4:** Bowel incontinence *N* = 7.

Item discussed	*N* = 7
Number of years have had bowel incontinence	Mean 5 years Median 7 years Range 1–10 SD 1.8
Do you experience bowel incontinence during the night?	Yes 5 adults No 1 adult Don't know 2 adults
How much does bowel incontinence interfere with your daily life? (Likert scale between 0 (not at all) and 10 (a great deal))	3 on scale, 1 adult 7 on scale, 1 adult 8 on scale, 3 adults 10 on scale, 2 adults

### 3.4 Toileting Issues

The POTI assessment checklist results are presented in Table 5. The seven most common toileting issues, which were reported for more than half of the sample, were as follows: daytime toileting accidents (20, 91%); wet underwear in the past month (19, 86%); not independent in self‐care (18, 82%); night‐time toilet accidents (16, 73%); requires encouragement to go to the toilet (16, 73%); does not stop activity to use restroom (13, 59%); and has strong urge before toileting accident (12, 55%).

**TABLE 5:** POTI Assessment Checklist (adapted for use with people with ID).

Item number and description	Yes	No	Don't know
1. Has daytime toileting accidents	20 (91%)	2 (9%)	0 (0%)
2. Has night‐time toileting accidents	16 (73%)	6 (27%)	0 (0%)
3. Has wet underwear in the past month	19 (86%)	3 (14%)	0 (0%)
4. Has soiled underwear in the last month	11 (50%)	11 (50%)	0 (0%)
5. Does not experience bowel movement once every 3 days	0 (0%)	22 (100%)	0 (0%)
6. Lost toileting skills	9 (41%)	13 (59%)	0 (0%)
7. Notice smears in underpants	8 (36%)	14 (64%)	0 (0%)
8. Others complain of odour	0 (0%)	22 (100%)	0 (0%)
9. Had constipation prior to age 3 months	0 (0%)	17 (77%)	5 (23%)
10. Has strong urge before toileting accident	12 (55%)	6 (27%)	4 (18%)
11. Has difficulty starting to urinate	3 (14%)	17 (77%)	2 (9%)
12. Has excessive posturination dribbling	2 (9%)	17 (77%)	3 (14%)
13. Requires to use laxatives	10 (45%)	12 (55%)	0 (0%)
14. Refuses to use the restroom	2 (9%)	20 (91%)	0 (0%)
15. Hides wet clothes	2 (9%)	20 (91%)	0 (0%)
16. Does not stop activity to use restroom	13 (59%)	9 (41%)	0 (0%)
17. Complains of stomach aches	4 (18%)	17 (77%)	1 (5%)
18. Does not communicate need to use restroom	11 (50%)	11 (50%)	0 (0%)
19. Hides soiled clothes	1 (5%)	21 (95%)	0 (0%)
20. Gets teased about accidents/odour	0 (0%)	22 (100%)	0 (0%)
21. Does not let caregiver know about wet/soiled clothes	4 (18%)	18 (82%)	0 (0%)
22. Has lack of appetite	1 (5%)	21 (95%)	0 (0%)
23. Motor problems interfere with toileting	9 (41%)	13 (59%)	0 (0%)
24. Not independent in self‐care	18 (82%)	4 (18%)	0 (0%)
25. Requires encouragement to toilet	16 (73%)	5 (23%)	1 (4%)
26. Exhibits problem behaviours when asked to toilet	3 (14%)	19 (86%)	0 (0%)
27. Attempts to toilet even if they have not defecated in 24–48 h	0 (0%)	22 (100%)	0 (0%)
28. Gets yelled at to use the restroom	0 (0%)	22 (100%)	0 (0%)
29. Does not become upset after accident	7 (32%)	14 (64%)	1 (4%)
30. Frequently wakes to toilet	5 (23%)	16 (73%)	1 (4%)

Participants' own descriptions of their incontinence identified a further 10 toileting issues. These were refuses to wear or removes pads (2, 9%); anxiety/distress around voiding into a pad (2, 9%); urinates and defecates on the floor (2, 9%); urine retention (previous nurse assessment following a bladder scan) (1, 4%); fear of falling (1, 4%); excessive fluid intake (1, 4%); does not aim for the toilet bowl when standing to urinate (1, 4%); struggles to remove lower garments to use the toilet (1, 4%); averse to sitting on a toilet to defecate (1, 4%); and not being prescribed enough pads for incontinence (1, 4%).

## 4 Discussion

Incontinence is commonly experienced by adults with ID, yet this is an underresearched area. This study highlights a number of issues which require attention, to develop an evidence‐base of best practice for adults with ID and incontinence; from developing an understanding of the health conditions and risk factors associated with incontinence in adults with ID, to codeveloping usable methods of assessment to inform management and treatment.

### 4.1 Factors Associated With Incontinence

The majority of the sample were women (13, 59%), with a mean age of 43 years, and 10 adults (45%) were reported as having severe or profound ID. Participants had been experiencing UI for an average of 8 years, and seven (32%) were also experiencing BI (for an average of 5 years). Only three adults (14%) had been incontinent their whole life (never achieved toilet training). In terms of known factors associated with UI (Bardsley 2016), all were identified as being common in this sample, with the exception of obesity as weight was not measured. All of these factors are potentially modifiable, to reduce or prevent future instances of UI in individuals with ID. This study demonstrates that further research on prevalence and risk factors for UTIs in adults with ID is warranted.

In terms of known health conditions, which can cause UI and/or BI, four adults (23%) did have cerebral palsy, and one adult (4%) had been diagnosed with Cauda Equina syndrome. In addition, four adults (18%) have type 2 diabetes, which can contribute towards UI (Lifford et al. 2005). The incontinence and toileting issues assessment conducted in this study was noninvasive, but a previous case study, which diagnosed Cauda Equina syndrome in one person with Down syndrome (Nair et al. 2015), has highlighted the importance of being able to detect serious physical reasons for incontinence in people with ID, and not at consequence of diagnostic overshadowing, attributing their incontinence to their ID or adaptive functioning. This stresses the importance of including the person's medical team in the assessment, when the person has developed incontinence, and other causes, such as presence of a UTI, have been ruled out.

### 4.2 Incontinence Assessments

Completing incontinence assessments with adults with ID and their supporters, with standard assessments developed with and for the general population, proved problematic. This was because of some questions' wording being too difficult to understand and/or the adult with ID not being able to answer subjectively, or convey whether or not they experienced the issue to their supporter. For example, for 4 out of 7 questions in the IPSS checklist, between 4 (18%) and 12 (55%) ‘don't know’ responses were given. There was also inconsistent reporting across similar questioning. For example, whilst 20 (92%) said they experienced a strong urge to urinate, elsewhere in the assessment the figure was 12 (55%). This demonstrates a need to develop more accessible and reliable incontinence assessment materials with and for people with ID and their supporters. The importance of coproduction of physical and mental health assessments with and for adults with ID is evidenced in the literature: from cervical screening for women with ID (Bateson et al. 2024) to developing assessment to manage risks for adults with ID in forensic settings (Morris et al. 2021).

### 4.3 Physical Measures

Six adults in this sample completed bladders scans, none of which were found to have PVUR. However, 18 adults were untested; one of whom had been diagnosed with PVUR following a bladder scan completed by a nurse. Unlike the De Waal et al. (2009) study, the majority of this sample refused bladder scans. Perhaps being offered bladder scans via a health professional better known to the person could help this.

Fifty per cent of those tested were found to have a probable or possible UTI, and 50% of the whole sample had been treated for a UTI within the previous 12 months. This finding highlights the importance of regular screening for UTIs in adults with ID and incontinence.

### 4.4 Bowel Assessment

The majority (17 adults, 77%) did pass a bowel movement on a daily basis, but seven adults (32%) did experience pain or discomfort when passing a bowel movement. Thirteen adults (59%) have constipation, which was the most commonly reported health condition reported for this sample, other than UI. Constipation is commonly experienced by adults with ID (Laugharne et al. 2024; Fitzpatrick et al. 2023) and can cause or exasperate UI (Bardsley 2016).

### 4.5 Toileting Issues

The POTI assessment checklist was useful for identifying the toileting issues experienced by adults with ID, the results of which were largely similar than those previously reported for other adults with ID (Matson, Horovitz, and Sipes 2011a). However, additional open‐ended questions identified a further 10 toileting issues, not previously reported elsewhere. It is important to include open‐ended questions in individual toileting issues assessment to identify all possible toileting issues.

### 4.6 Strengths and Limitations

The main strength of this study is that it provides a current snapshot, describing incontinence and toileting issues experienced by adults with ID and UI, with or without BI. Previous literature is dated. Detailed information about incontinence, associated factors and toileting issues amongst adults with ID are all necessary for developing strategies and interventions to manage or treat such. The main limitation is the small but exploratory sample.

## 5 Conclusion

Any assumption that incontinence may be attributable to a person's ID would be false, as the majority of this sample (19 adults, 86%) had previously been continent, and developed incontinence during adulthood. Whilst reflex and functional UI may be more prevalent amongst people with ID, symptoms associated with other types of UI (e.g., sudden urge to urinate) are also evident.

There is an urgent need to coproduce more accessible and reliable incontinence assessment measures with and for adults with ID and their supporters. These assessments should also play close attention to existing health conditions as possible reasons for incontinence and factors associated with incontinence. These factors are potentially modifiable, for developing strategies and interventions to reduce or prevent further instances of incontinence in this group.

In terms of toileting issues, it is clear that some individuals with ID and incontinence require support to use a toilet (e.g., not being independent in self‐care, or requiring reminders to use a toilet or stop an activity to use a toilet). Strategies and interventions should be in place to ensure adults with ID have adequate levels of support to meet their toileting needs, and be reviewed regularly, as their needs are likely to change over time. In addition, assessment of toileting needs should also include open‐ended questions (e.g., ‘Please describe any toileting issues, needs or worries you may have’), to ensure the assessment is tailored to the individual person concerned.

## Conflicts of Interest

The authors declare no conflicts of interest.

## Data Availability

Data are available on request from the authors.
